# Effects of Airflow Ultrafine-Grinding on the Physicochemical Characteristics of Tartary Buckwheat Powder

**DOI:** 10.3390/molecules26195841

**Published:** 2021-09-26

**Authors:** Qinglian Xu, Faying Zheng, Xiaotong Cao, Ping Yang, Yage Xing, Ping Zhang, Hong Liu, Guangchao Zhou, Xiaocui Liu, Xiufang Bi

**Affiliations:** 1 Key Laboratory of Grain and Oil Processing and Food Safety of Sichuan Province, College of Food and Bioengineering, Xihua University, Chengdu 610039, China; xuqinglian01@163.com (Q.X.); zfyasdzxc2021@163.com (F.Z.); cxc442278103@163.com (X.C.); yangp960119@163.com (P.Y.); gyliuhong@126.com (H.L.); xiaocuiliu777@126.com (X.L.); bxf1221@163.com (X.B.); 2 Huantai Biotechnology Co., Ltd., Chengdu 610225, China; htzp2019@163.com (P.Z.); zhou_423yan@163.com (G.Z.)

**Keywords:** Tartary buckwheat powder, airflow ultrafine-grinding, grinding pressure, particle size, chemical compositions, morphology

## Abstract

Five different ultrafine milled flours (UMFs) were prepared from Tartary buckwheat via airflow ultrafine-grinding at different grinding pressures. The airflow ultrafine-grinding resulted in marked differences in particle size (from 100 to 10 μm). The UMFs were all brighter in appearance (higher L*) than Tartary buckwheat common flour (TBCF). Illustrated by the example of 70 °C, the UMFs were also found to have a greater water holding capacity (from 4.42 g/g to 5.24 g/g), water solubility (from 12.57% to 14.10%), and water solubility index (from 5.11% to 6.10%). Moreover, as the particle sizes reduced, the moisture content decreased (from 10.05 g/100 g DW to 7.66 g/100 g DW), as did the total starch content (from 68.88 g/100 g DW to 58.24 g/100 g DW) and the protein content (from 13.16% to 12.04%). However, the grinding process was also found to have negative effects on the mineral content of the Tartary buckwheat. Additionally, several substantial variations were found in their hydration properties along with grinding pressure changes in the differently ground UMFs. Consequently, fine Tartary buckwheat powders of a bright yellow color, with superior food processing properties, were prepared in this study by airflow ultrafine-grinding.

## 1. Introduction

Buckwheat is a gluten-free pseudocereal belonging to the Polygonaceae family of plants. It contains protein of a high nutritional value, dietary fiber, vitamins, and minerals [1,2,3]. Tartary buckwheat (or bitter buckwheat) is the most commonly cultivated species, which has a higher concentration of bioactive compounds, such as flavonoids and phenolics [4,5,6]. Tartary buckwheat is, therefore, an important coarse cereal and is expected to become the target of many future planting industries [7]. In China, Tartary buckwheat is cultivated at high altitude, mountainous regions, and harsh climatic conditions [8]. In the Liangshan Yi Autonomous Prefecture, located in the Yunnan-Guizhou Plateau, the cultivation of Tartary buckwheat is an important source of income for local farmers in the high mountains and hilly areas, where other major crops may fail. By 2019, Tartary buckwheat plantations covered more than 2660 square kilometers in the Liangshan Prefecture.

In comparison to buckwheat, Tartary buckwheat has attracted more food science research interest due to its unique chemical composition and high efficiency as a functional food, including its anti-oxidative, anti-cancer, anti-hypertension, and anti-diabetic properties [9]. Tartary buckwheat is the crop that provides seven nutrients, with quantities of sugar, protein, fat, minerals, fiber, vitamins, and water, and its outstanding health and nutritional properties, thus endow it with a high edible value [10]. Furthermore, Tartary buckwheat extract exhibits high levels of antioxidant activity. As an exogenous antioxidant, it can promote or interact with endogenous antioxidants to form a cooperative network of cellular antioxidants [11]. One study found that the extract could increase the contribution of exercise-induced oxidative stress, thereby improving the physiological condition of humans [12]. The development of Tartary buckwheat as a beneficial nutritional supplement has thus far been suppressed by its bitter flavor and poor palatability, however, with the application of new food processing methods, products such as Tartary buckwheat-enriched biscuits, noodles, and beverages have been introduced and are increasing in popularity among consumers [13,14,15,16,17].

Superfine grinding technology, which is developing rapidly in food processing, can produce powder with superior properties to conventional particles and it is, thus, being used increasingly with a variety of food materials to improve the quality of powder [18]. Micronization is the process of reducing the average diameter of a solid material’s particles [19,20]. Superfine grinding methods including airflow grinding, liquid flow grinding, low-temperature grinding, ball milling, ultrasonic disintegrator grinding, etc. [21]. To date, this ultrafine grinding technology has been applied in biotechnology and to achieve various foodstuffs, such as ginger powder, mushroom powder, green tea powder, hull-less barley bran, Agrocybe chaxingu Huang powder, and whole-wheat powder [22,23,24,25,26,27]. Wu et al. found that ultrafine grinding improved the solubility, oil-holding capacity, and brightness of Panax notoginseng powder. The contents of total saponins, minerals, phenols, and flavonoids were highest, and the antioxidant activity was the best, in the smallest particles of the Panax notoginseng powder [28]. In another study, the ultrafine powder of pomegranate peel showed strong specific surface energy, significant fluidity, and superior water-holding capacity, water solubility, polyphenols and flavonoids release, and DPPH radical scavenging activity, and it could be used as a new value-added product to provide health benefits in food processing because of its excellent dispersibility and dissolution [29]. These studies suggest that ultrafine grinding is a useful tool for producing ultrafine powders with good surface properties such as dispersion and solubility [30]. Moreover, it has been revealed that the physical and functional properties of buckwheat powder are affected differently by different milling methods, which thus affect the properties of products [31,32]. However, very limited information is available on the effects of airflow ultrafine-grinding on the physical characteristics of Tartary buckwheat powder.

The aim of this work is, thus, to investigate the application of airflow ultrafine-grinding technology on Tartary buckwheat. In this paper, five different ultrafine milled flours (UMFs) were prepared from Tartary buckwheat via airflow ultrafine-grinding at different grinding pressures. The particle size analysis and morphological observation were firstly conducted. Furthermore, the physicochemical characteristics of obtained different particles were analyzed, including chemical composition, hydration properties, and mineral content, in comparison to those of commonly ground Tartary buckwheat particles.

## 2. Results and Discussion

### 2.1. Particle Size Analysis and Morphological Observation

**Figure 1:**
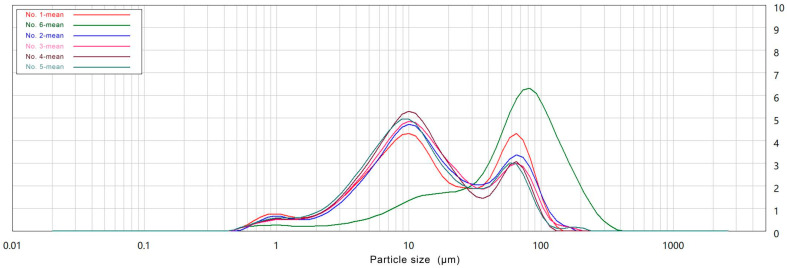
Particle size distribution of different Tartary buckwheat flour via airflow ultrafine-grinding at different grinding pressures. (No. 1: Tartary buckwheat ultrafinemilled flour No. 1; No. 2: Tartary buckwheat ultrafinemilled flour No. 2; No. 3: Tartary buckwheat ultrafinemilled flour No. 3; No. 4: Tartary buckwheat ultrafinemilled flour No. 4; No. 5: Tartary buckwheat ultrafinemilled flour No. 5; No. 6: Tartary buckwheat common flour No. 6).

Analysis of the distribution of the UMF particle sizes obtained via the airflow ultrafine-grinding methods (Figure 1) showed them to be between 10 μm and 80 μm, demonstrating them to be within the size range of ultrafine powder [33].Furthermore, the Dv values of the Tartary buckwheat samples are summarized in Table 1. Generally, D10, D50, and D90 are used to characterize particle size distributions, and are the equivalent volume diameters at 10%, 50% and 90% of the cumulative volume, respectively [34]. It was found that the mean particle size of the TBCF was 100 μm, which was almost three times, five times, and two times that of the UMF for D10, D50, and D90, respectively. The D50 values of the UMFs and the TBCF were 10.97 μm, 11.19 μm, 12.24 μm, 13.18 μm, 13.07 μm, and 61.84 μm. Additionally, with increases in grinding pressure, the particle sizes of the ultrafine powder decreased; however, there were no significant (*p* > 0.05) differences between the samples at a grinding pressure at 0.1 MPa (No. 1 and No. 2), and when the grinding pressure changed by 0.2 MPa, the particle sizes of the UMF showed a significant (*p* < 0.05) decline. These results indicated that the increase in grinding pressure produced a finer UMF comprised of small particles, while the uniformity of the powder was also improved.

**Figure 2:**
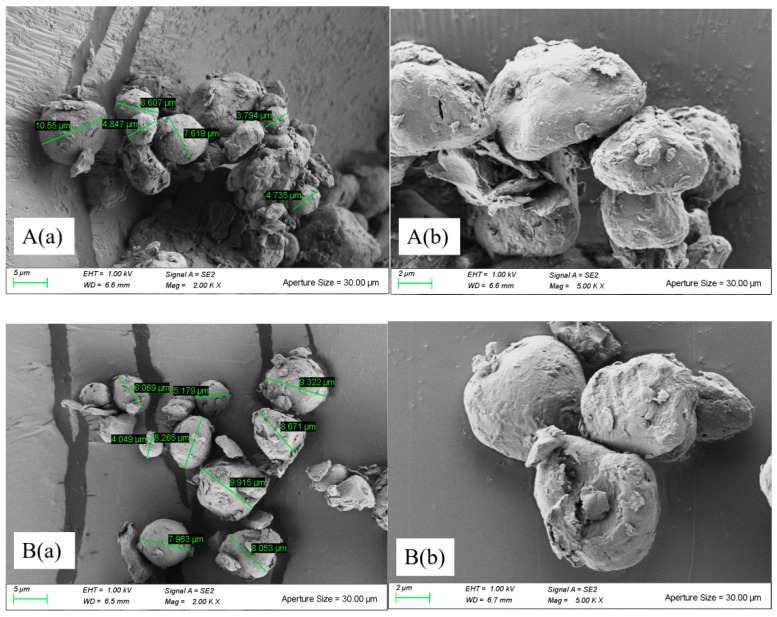

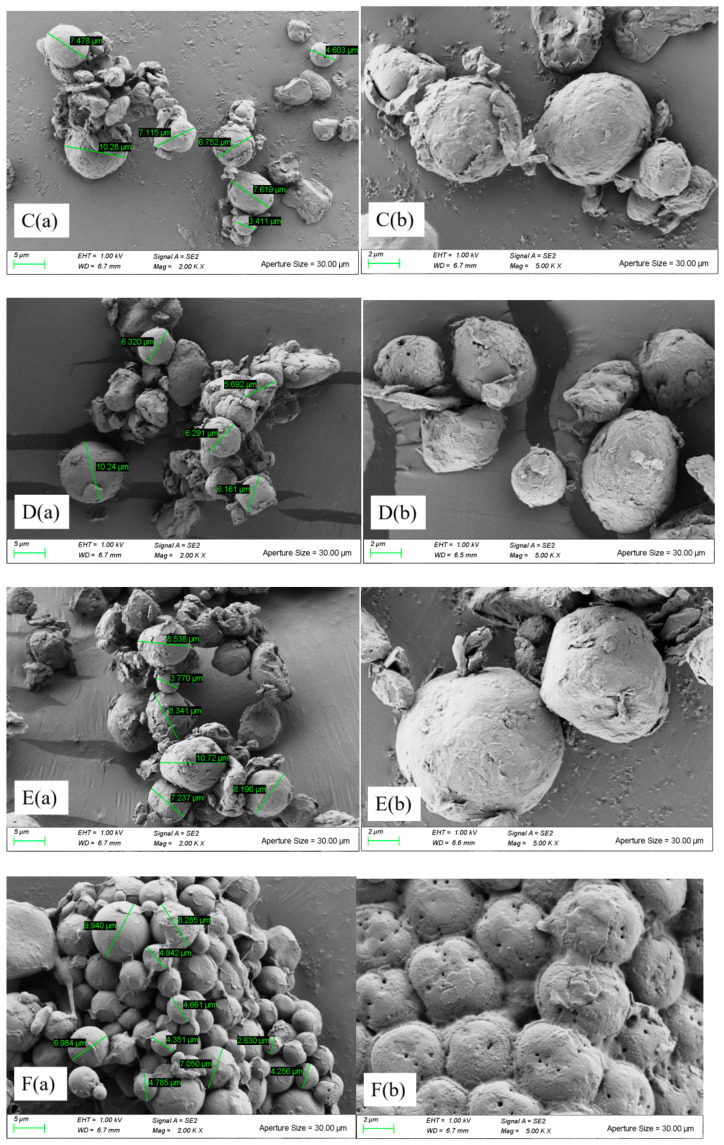
Scanning electron microscopy observation of different Tartary buckwheat flour via airflow ultrafine-grinding at different grinding pressures. (Tartary buckwheat ultrafinemilled flour No. 1 (**A**); Tartary buckwheat ultrafinemilled flour No. 2 (**B**); Tartary buckwheat ultrafinemilled flour No. 3 (**C**); Tartary buckwheat ultrafinemilled flour No. 4 (**D**); Tartary buckwheat ultrafinemilled flour No. 5 (**E**); Tartary buckwheat common flour No. 6 (**F**); (**a**) is magnified 2000 times and (**b**) 5000 times.)

To further understand the morphological properties of Tartary buckwheat powders, the samples in this study were analyzed using SEM and differences were found in the shapes, sizes, and granule surfaces of the powders with and without the ultrafine milling treatment. As shown in Figure 2, the treated Tartary buckwheat flour particles were spherical, oval, regular, and polygonal in shape, and were also highly uniform. In addition, the surface roughness of No. 1 (grinding pressure at 0.8 MPa) was found to be greater than that of the other five superfine powders (Figure 2A), which were seriously damaged because of mechanical force, with rough surfaces and a large number of residual fibrous fragments and protein fragments. However, the images in Figure 2F(b) reveal that the powder particles of the TBCF were uniform, arranged in an orderly manner with more pores. Moreover, the particle sizes of the TBCF sample were significantly larger than those of the UMFs, as verified by morphology observation (Figure 2F). This suggested that airflow ultrafine-grinding influenced the morphological characteristics of the Tartary buckwheat flour, increasing its surface area and leading to the increase in surface properties. Moreover, the changes in the particle size of the Tartary buckwheat flour had significant effects on its physico-chemical properties.

Ultrafine grinding technology can improve the structural characteristics of Tartary buckwheat powders by reducing their particle size, and the morphology of Tartary buckwheat flour is also changed by different grinding pressures [35]. In our study, the results indicated that superfine pulverization using a mini-type airflow pulverization instrument could reduce the sizes of the Tartary buckwheat powder particles. The ultrafine pulverization process produces more energy with higher reduction efficiency, making it easier for Tartary buckwheat flour to be shaped into a powder of smaller particle sizes, while the rough surface of the powder is created by the strong grinding force, which destroys the integrity of the starch granules during the ultrafine grinding process, producing starch granules with smaller fragments [36]. The UMF, with its narrower particle size distribution and more even particle size obtained via airflow ultrafine-grinding treatments, is more easily processed to significantly improve the quality and taste of products. Similar results were observed in red grape pomace powders (Zhao et al., 2015) and whole-wheat flour (Niu et al., 2014), while contrary results were discovered in red grape pomace powders (Zhao et al., 2015), in which superfine grinding was found to smooth the surface [27,37]. Such differences may be caused by varying experimental conditions, such as working frequency, feeding velocity, feeding pressure, and grinding pressure. In our experiment, the series of grinding pressures were set before experimentation began.

Ultrafine grinding was applied in this study. In general, the higher the pressure, the smaller the particle size [26]. As expected, the particle size decreased with increasing grinding pressure; however, there were no significant differences in the particle sizes of the No. 1 and No. 2 samples, indicating that grinding pressure may not have had much effect on the smaller powders. This result is consistent with the findings of Zhang et al., in which the powder of Agrocybe chaxingu Huang was prepared using ultrafine grinding [26]. The results showed that the particle size of the powder decreased from 110 μm to 20 μm when the grinding pressure was increased from 0.3 MPa to 0.4 MPa. However, when the grinding pressure continued to increase from 0.1 MPa to 0.5 MPa, the particle sizes of the Agrocybe chaxingu Huang hardly changed. While a substantial increase in grinding pressure did indeed change the particle size of the Tartary buckwheat flour in this study, it should also be noted that the smaller the particles, the less significant the impact of any further increase in grinding pressure on their size.

### 2.2. Color Difference Analysis

Color and particle size are important qualities in food powders. Color is an important parameter for most food products and is usually a consideration for consumers in their evaluation and selection of products. The investigation by Hu et al. indicated that particle size had a significant effect on green tea powders [24]. The influence of various particle sizes on the color of Tartary buckwheat flour in this study is presented in Table 2, in which ‘L’ expresses brightness, while ‘a’ and ‘b’ are chromaticity coordinates, with +a indicating the red direction, −a the green direction, +b the yellow direction, and −b the blue direction [38]. As illustrated by No. 5 in Table 2, compared to the TBCF sample, ‘L’ values increased significantly (*p* < 0.05) from 88.95 to 93.37, whereas ‘a’ and ‘b’ values decreased markedly (*p* < 0.05) from 2.36 to 1.70, and 22.07 to 17.29, respectively. These results indicate that ultrafine grinding improved the color of the Tartary buckwheat flour, making it appear brighter, while the appearance of the TBCF was a dim yellow in comparison. Moreover, as shown in Table 3, ‘L’ increased via the treatment No. 5 to No. 3 but decreased from treatment No. 3 to No. 2, then finally increased from No. 2 to No. 1. By contrast, ‘a’ and ‘b’ showed the opposite trend. In addition, with increasing grinding pressure, No. 1 expressed the highest level of brightness, indicating that the parameter of grinding pressure had an effect on the brightness of the Tartary buckwheat.

In this investigation work, the brightness of Tartary buckwheat flour was negatively correlated with particle size. The smaller the particle size, the greater the relative surface area, the better the reflective effect, and the greater the brightness value. Therefore, Tartary buckwheat flour with the highest level of brightness was obtained by the airflow ultrafine-grinding treatments. Moreover, the grinding treatments’ improvement of the color of the Tartary buckwheat may be due to the fact that surface area increased with the decrease in particle size, and the internal structure of the cellulose and hemicellulose were exposed, which affected the color of the powder [39]. The same results were obtained in the experiments of Hu and Li, in which the colors of green tea powder and soybean residue powder, respectively, changed significantly with decreases in particle size during superfine grinding [24,36]. Huang et al. also found that the superfine powder of the Moringa oleifera leaf was obviously brighter than the Moringa leaf powder of millimeter grade, indicating that particle size and superfine grinding technology had a significant effect on the brightness of those powders [40]. Notably, in this study, neither the brightness nor the chromaticity coordinates of the Tartary buckwheat flour were positively correlated with grinding pressure. Color changes could be related to the heat in the UMF process. Since the smaller particles are subjected to more mechanical and high temperature damage during grinding, the pigment in the Tartary buckwheat powder was degraded, resulting in increased whiteness. Overall, UMF of smaller particle sizes was relatively brighter, and either more green or more blue in color. Importantly, since the color of a powder has a great influence on its processed products, a compound product of Tartary buckwheat may be more easily accepted by consumers because of its brightness [41].

### 2.3. Chemical Composition of Tartary Buckwheat Flour

The effects of airflow ultrafine-grinding on the chemical compositions of the flour samples are summarized in Table 3, in which it is evident that they varied greatly. The maximum UMF moisture content (8.22 g/100 g) was significantly (*p* < 0.05) lower than that of the TBCF (10.05 g/100 g). A significant difference (*p* < 0.05) was also observed in the protein content among all samples. The crude fiber content of the UMF could not be determined because of their particle size; thus, there was no significant difference (*p* > 0.05) in the crude fat content of the UMF and TBCF. Moreover, the total starch content, which accounted for the proportion of TBCF, was approximately 84.55% in the UMF. Furthermore, changes in the mineral content of the Tartary buckwheat flour were evaluated after the airflow ultrafine-grinding treatments. As shown in Table 3, Ca content in the UMF was significantly (*p* < 0.05) lower than that in the TBCF, as was the content of Fe, with the exception of No. 1. There were no obvious increasing or decreasing trends in the Mn, Zn, or Cu contents among the UMFs treated by different grinding pressures. However, the contents of protein and total starch decreased with increasing grinding pressure, from 13.16 to 12.04% and from 67.64 g/100 g DW to 58.24 g/100 g DW, respectively.

The variations in the composition of the samples may be attributed to different operating conditions. Tartary buckwheat flour is influenced by the heat produced by the machine during milling and it is, therefore, likely that there would be changes in the contents of fat, starch, protein, and moisture during the grinding process. These results are consistent with a study by Liu et al., who found that the quality of protein and lipids could be changed by high temperatures during milling [32]. In addition, by studying the effects of superfine grinding on the quality characteristics of whole-wheat flour and its raw noodle product, Niu found that grinding treatments could induce the physical transformation of starch granules, including a reduction in the starch crystal area and an increase in starch damage, leading to changes in the properties of the starch [27]. Here, in summary, the mineral content of the Tartary buckwheat powder decreased after airflow ultrafine-grinding, and the Ca and Fe contents changed significantly, possibly because the friction of the materials bouncing off each other caused heat. Similar results were found by Liu et al. in their study of the effects of grinding methods on the chemical composition and antioxidant capacity of Tartary buckwheat powder, in which they compared the effects of four different grinding methods on the mineral content. The results showed that the mineral content of the Tartary buckwheat powder obtained by stone grinding was significantly higher than that of the ultrafine powder, almost triple that of roller milling and quadruple that of wet grinding [32].

### 2.4. Hydration Properties

#### 2.4.1. Water Holding Capacity Analysis

**Figure 3:**
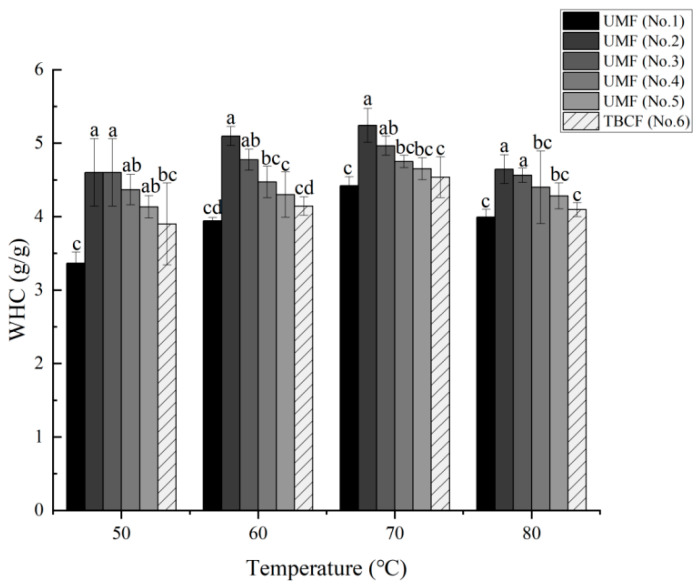
Effects of airflow ultrafine-grinding on the water holding capacity in Tartary buckwheat flour. The values represented by the different letters are significantly different at *p* < 0.05 compared with each other among the same temperature.

The water holding capacity (WHC) of the five different particle-sized UMFs and TBCF sample are shown in Figure 3. It was initially discovered that the WHC increased with the decreasing size of Tartary buckwheat particles. The WHC increased at first and then decreased within the measured temperature range (from 50 °C to 80 °C) and reached its highest level at 70 °C, at which it was recorded in the following order: 5.24 g/g (No. 2) > 4.97 g/g (No. 3) > 4.75 g/g (No. 4) > 4.65 g/g (No. 5) > 4.54 g/g (No. 6) > 4.42 g/g (No. 1). With the exception of No. 1, the WHC was higher in all UMFs than that of the TBCF at all temperatures, indicating that the WHC of Tartary buckwheat flour was improved during airflow ultrafine-grinding. In addition, with increasing grinding pressure, the WHC of the ultrafine powders increased at the same temperature except for sample No. 1. This result may have been because, when the particle size decreased to a certain extent, the gaps between them also decreased, resulting in a lower ability of the powder to retain water. More importantly, with the increase in temperature, the WHC of the superfine powder increased significantly, indicating that it had improved hydrophilic ability, which can prevent the loss of water and delay the aging of starch in the hot process.

WHC refers to the quantity of water that is bound to the fibers without the application of any external force (except for gravity and atmospheric pressure) [42]. Airflow ultrafine-grinding technology produces powders with a greater WHC than TBCF because the grinding process lowers the interfacial tension and exposes more polar groups, surface area, and water-binding sites to the surrounding water [43]. The significant improvement in the WHC of the UMF may be due to changes in the surface properties of the superfine powders, such as the increase in surface area, gaps, and liquid holding space. Zhao et al. found that superfine grinding could alter or destroy the internal structure of ginger, thus resulting in the exposure of its hydrophilic groups and subsequent easier integration with water, which eventually increased its WHC [22]. Similar results were found in the studies of Astragalus membranaceus powder, pear pomace powder, and Lycium ruthenicum Murray powder [35,44,45,46].

#### 2.4.2. Swelling Power and Solubility Analysis

**Figure 4:**
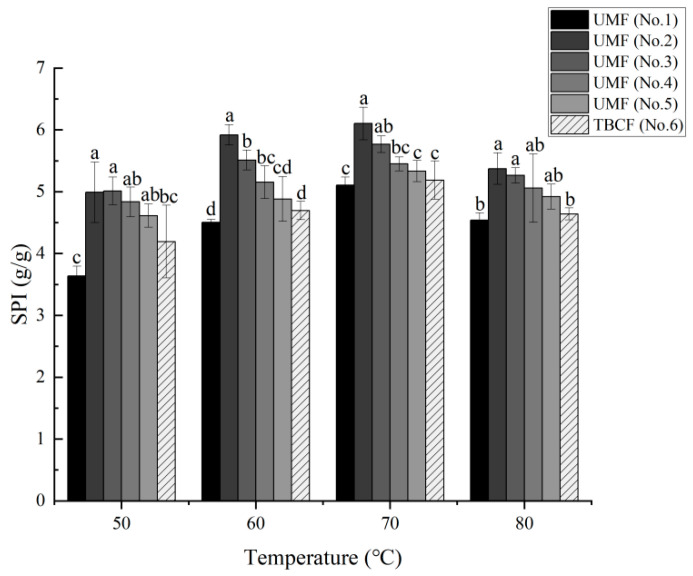
Effects of airflow ultrafine-grinding on the swelling power in Tartary buckwheat flour. The values represented by the different letters are significantly different at *p* < 0.05 compared with each other among the same temperature.

**Figure 5:**
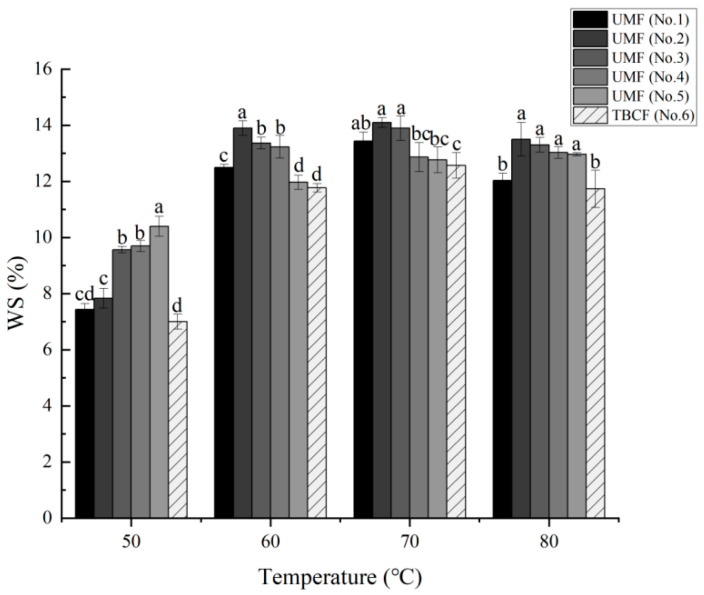
Effects of airflow ultrafine-grinding on the water solubility in Tartary buckwheat flour. The values represented by the different letters are significantly different at *p* < 0.05 compared with each other among the same temperature.

Solubility and the degree of expansion during steaming treatment reflect the quality of Tartary buckwheat flour. The swelling power of TBCF and ultrafine Tartary buckwheat flour at different temperatures (from 50 °C to 90 °C) are shown in Figure 4. At the same temperature, almost all UMF samples exhibited a higher SPI than that of the TBCF. When the temperature reached 70 °C, swelling power reached its highest levels, with indexes recorded in the following order: 6.10% (No. 2) > 5.77% (No. 3) > 5.45% (No. 4) > 5.33% (No. 5) > 5.19% (No. 6) > 5.11% (No. 1). Furthermore, for the samples from No. 5 to No. 2, with increasing grinding pressure, the swelling power of the samples increased at the same temperature. The results showed that the superfine milling significantly changed the swelling power of the Tartary buckwheat flour and the prevention of water dispersion incapacitation was also enhanced. Similar results were also obtained for WS, shown in Figure 5. The WS of the Tartary buckwheat flour increased at first and then decreased slightly within the measured temperature range (from 50 °C to 80 °C), reaching its highest level at 70 °C, at which WS was recorded in the following order: 14.10% (No. 2) > 13.90% (No. 3) > 13.43% (No. 1) > 12.87% (No. 4) > 12.77% (No. 5) > 12.57% (No. 6). These results, thus, also showed that the WS of the Tartary buckwheat flours were improved by airflow ultrafine-grinding technology.

Water solubility and swelling power provide a measure of the degree of reciprocal action between starch chains in the amorphous and crystalline domains [47,48,49]. In our study, the swelling power of the ultrafine powder was found to be generally higher than that of the TBCF, possibly indicating that the starch structure of the Tartary buckwheat powder was destroyed, and that the content of amylopectin had increased in the process of airflow ultrafine-grinding [50]. This is similar to the results of Huang et al., in which the double helix structure of starch was destroyed by ultrafine grinding and the molecular structure of amylose was depolymerized [51]. In addition, water molecules can easily enter the inner regions of starch particles after ultrafine grinding, which could reduce the interaction between amylose and amylopectin molecules [52]. This may explain the higher swelling power of Tartary buckwheat after ultrafine grinding compared with that of common buckwheat. Similar observations have been made in superfine powders of buckwheat starch [51]. In terms of WS, the index increased as the size of the Tartary buckwheat particles decreased, which may be attributed to the larger surface area and high charge density [53]. Furthermore, the porosity of the powders was increased after superfine grinding, thus raising the hydration rate and bioavailability of components [48]. Similar phenomena were observed in the study of Vaccinium bracteatum Thunb leaves powder. However, these results also indicate that the paste phenomenon is more obvious in UMF processing.

It is worth noting that not all samples showed higher hydration properties after superfine grinding at the same temperature, with the exception of a No. 1 sample (prepared under the maximum pressure of 0.8 MPa). Generally speaking, with the increase in grinding pressure, the particle sizes in the ultrafine powder decreased. This is mainly because, at a certain range, the increase in grinding pressure improves the velocity of airflow at the outlet of the nozzle, causing the material to gain more kinetic energy. When particle sizes decrease to a certain value, the particles agglomerate with the increase in the powder surface energy. This may explain why the hydration properties of No. 1 sample were almost the same as those of the TBCF after ultrafine grinding. Therefore, the surface properties of powder may change dramatically after superfine grinding under high crushing pressure [54].

## 3. Materials and Methods

### 3.1. Main Material

The Tartary buckwheat powder used in this work was produced by Huantai Biotechnology Co., Ltd. (Sichuan, China). Sodium hydroxide, petroleum ether, sulfuric acid, hydrochloric acid, anhydrous ethanol, and nitric acid were purchased from Cologne Co., Ltd. (Chengdu, China), among which the nitric acid was of guaranteed grade. Iron (Fe), calcium (Ca), zinc (Zn), copper (Cu), and other standard solutions were obtained from the National Research Center for Standard Materials.

### 3.2. Preparation of Tartary Buckwheat Ultrafine Milled Flours

Tartary buckwheat common flour (TBCF) was passed through a 120-mesh sieve, and then dried in a constant temperature blast oven at 50 °C for 1 h. The TBCF was further pulverized using an airflow pulverization instrument (YQ50-1, Saishan Powder Machinery Manufacturing Co., Ltd., Shanghai, China), and the grinding pressure was variously regulated to 0.4 MPa, 0.5 MPa, 0.6 MPa, 0.7 MPa, and 0.8 MPa, finally resulting in five different ultrafine milled flours (UMF), correspondingly labeled No. 5, No. 4, No. 3, No. 2, and No. 1, respectively. The TBCF was used as the control sample, labeled No. 6.

### 3.3. Determination of the Chemical Compositions

Moisture, protein, and crude fiber in all samples were determined using the method described by Pandord, with some modifications [55]. Moisture content was determined after drying the samples at 105 °C for 4 h in an air oven. Proximate analysis of protein content was performed using the Kjeldahl method, with a conversion factor of N × 6.25. Crude fiber was determined by the filtration method, in which defatted samples were boiled in concentrated sulfuric acid for 30 min, in potassium hydroxide (instead of sodium hydroxide) for 30 min, then dried and, finally, reduced to ash in a muffle furnace. In addition, the crude fat content was assayed as described by Weber, with slight modifications [56]. Crude fat was obtained via extraction from the samples with petroleum ether (instead of hexane) for 6 h in a Soxhlet apparatus, then dried in a water bath for 1 h at 100 °C, after which the weight differences of the samples were determined.

The starch in Tartary buckwheat (which accounts for approximately 70% of its content) is particularly important in determining the textural properties of its products [57]. In this study, the starch content was analyzed using the methods described by Senanayake with some modifications [58]. Briefly, a flour sample of 0.3 g was washed, first, with petroleum ether and then with ethanol, and the residue was subsequently filtrated in a solution of 250 mL water and 30 mL hydrochloric acid. The mixture was then heated for 2 h in a conical flask fitted with a reflux condenser, after which it was cooled and neutralized with sodium hydroxide (NaOH). The volume was increased to 500 mL with distilled water and the sugar formed was determined as dextrose using the Lane and Eynon method for the estimation of reducing sugars. The dextrose multiplied by 0.9 was taken as starch.

Minerals play an important role in the physiological functioning of the body, especially in the growth and metabolic regulation process [59]. The mineral content in the Tartary buckwheat flour was assayed as described by Özcan, with slight modifications [60]. Approximately 0.4 g dried and ground sample was placed in a digestion tank, to which 7 mL pure nitric acid (HNO3) was added. This mixture was then counteracted in a microwave digestion instrument at different temperatures. Mineral concentrations were determined via inductively coupled plasma (Avio 200 ICP Optical Emission Spectrometer; PerkinElmer Instrument Co., Ltd., Singapore).

### 3.4. Morphologies Observation of Tartary Buckwheat Flour

The particle size distribution of the Tartary buckwheat flour samples was determined using a laser particle size analyzer (Bettersizer 2600; Dandong Baxter Instrument Co., Ltd., Liaoning, China) in the wet method mode. In addition, the morphological characterization of Tartary buckwheat flour particles was performed on images acquired using a scanning electron microscope (SEM) (ZEISS Gemini 500; Xiangyan Co., Shanghai, China) at an accelerating voltage of 1.00 kV. A small amount of Tartary buckwheat flour adhered to the surface of the double-sided conductive carbon adhesive tape and an aurilave was used to remove those particles. The sample was then subjected to a gold spray treatment under vacuum conditions prior to testing.

### 3.5. Color Difference Analysis

The colors of the different Tartary buckwheat flour samples were determined using the VeriVide DigiEye system (Yunding International Trade Co., Ltd., Shanghai, China), and the device was calibrated with a standard white surface calibration plate. Six varieties of Tartary buckwheat flour were placed on a plate and then selected randomly. The L*, a*, and b* values for each sample, indicating lightness, redness (+)/(−) greenness, and yellowness (+)/(−) blueness, respectively, were recorded.

### 3.6. Determination of Hydration Properties

The water holding capacity (WHC), water solubility (WS) and water solubility index (SPI) of the Tartary buckwheat flour were measured using the method reported by Tsai, Li, and Lii [61]. Approximately 0.1 g of Tartary buckwheat flour and 10 mL of deionized water were placed together in a beaker and mixed thoroughly by an ultrasonic instrument. The beaker was kept in a water bath (at temperatures of 50 °C, 60 °C, 70 °C, and 80 °C) for 30 min, respectively, and then centrifuged for 20 min at 3000 r/m). The weighing bottle (m_1_) was weighed, and the collected supernatant was transferred to the pre-weighed weighing bottle, dried at 105 °C until the mass difference between the two was no more than 2 mg, and then weighed (m_2_). It was finally weighted (m_3_) after the supernatant had been discarded. The WHC, WS, and SPI were calculated by the following equation:

WHC = m_3_/0.1 (1)

WS = (m_2_ − m_1_)/0.1 × 100% (2)

SPI = m_3_/0.1 × (1 − WS) (3)

### 3.7. Statistical Analysis

All data were presented as the mean standard deviation of at least three replicates and analysis performed was one-way analysis of variance (ANOVA) followed by the Duncan’ s multiple comparison test using SPSS version 25.0 software.

## 4. Conclusions

In this study, airflow ultrafine-grinding treatment was found to exert significant influence on the physicochemical characteristics and mineral content of Tartary buckwheat powder. Smaller and brighter Tartary buckwheat powders, with greater food processing properties, were obtained after superfine grinding. With increases in grinding pressure, the particle sizes of the Tartary buckwheat powder decreased and reached equilibrium finally. Moreover, the superfine grinding process decreased moisture content, crude fat content, and protein content, which is beneficial for health and weight loss. The ultrafine Tartary buckwheat powder was found to retain the essential nutritional characteristics of the original Tartary buckwheat; however, its mineral content was affected and it is, therefore, important to consider both nutrition and the cost of the product when processing ultrafine Tartary buckwheat powder. Hydration properties were significantly improved in the superfine Tartary buckwheat powder and the increase in swelling power could benefit satiation; however, the higher water solubility also suggests that the superfine powder is more likely to create a soup paste during cooking. Further studies could be conducted on the polyphenol and lipid content obtained from Tartary buckwheat powders with different particle sizes. Superfine grinding technology could be used to broaden the application of Tartary buckwheat powders in functional foods.

## Figures and Tables

**Table 1:** Particle size distribution of Tartary buckwheat flour with different particle size ^1^.

Sample	D10 (μm)	D50 (μm)	D90 (μm)
No. 1	3.03 ± 0.01c	10.97 ± 0.04c	60.64 ± 0.44d
No. 2	3.21 ± 0.05bc	11.19 ± 0.15c	61.07 ± 0.59d
No. 3	3.33 ± 0.05bc	12.24 ± 0.18bc	67.49 ± 1.15c
No. 4	3.42 ± 0.04b	13.18 ± 0.07b	72.27 ± 0.38b
No. 5	2.96 ± 0.02c	13.07 ± 0.06b	71.83 ± 0.46b
No. 6	8.93 ± 0.48a	61.84 ± 2.36a	151.97 ± 2.20a

^1^ Values in the same column with different letters are significantly different at *p* < 0.05. (Abbreviations: No. 1: Tartary buckwheat ultrafinemilled flour No. 1; No. 2: Tartary buckwheat ultrafinemilled flour No. 2; No. 3: Tartary buckwheat ultrafinemilled flour No. 3; No. 4: Tartary buckwheat ultrafinemilled flour No. 4; No. 5: Tartary buckwheat ultrafinemilled flour No. 5; No. 6: Tartary buckwheat common flour No. 6).

**Table 2:** Color difference of different Tartary buckwheat flour obtained from airflow ultrafine-grinding ^1,2^.

Sample	L*	a*	b*
No. 1 (UMF)	95.52 ± 0.07a	1.02 ± 0.09d	14.90 ± 0.21d
No. 2 (UMF)	93.91 ± 0.07c	1.74 ± 0.04b	17.29 ± 0.11b
No. 3 (UMF)	95.32 ± 0.06a	1.47 ± 0.04c	15.57 ± 0.13c
No. 4 (UMF)	94.80 ± 0.03b	1.44 ± 0.03c	15.61 ± 0.20c
No. 5 (UMF)	93.37 ± 0.15d	1.70 ± 0.04b	17.29 ± 0.26b
No. 6 (TBCF)	88.95 ± 0.26e	2.36 ± 0.12a	22.07 ± 0.37a

^1^ Data are expressed as the mean ± standard deviation. Values in the same column with different letters are significantly different at *p* < 0.05. ^2^ The ‘L’ is the indicator of lightnesse-darkness; the ‘a’ is the indicator of greenness (when it is a minus value) and redness (plus value); the ‘b’ is the indicator of blueness (minus value) and yellowness (plus value).

**Table 3:** Effects of airflow ultrafine-grinding on the basic components in Tartary buckwheat flour ^1^.

Nutrient Contents	No. 1 (UMF)	No. 2 (UMF)	No. 3 (UMF)	No. 4 (UMF)	No. 5 (UMF)	No. 6 (TBCF)
Moisture (g/100gDW)	7.66 ± 0.02c	8.22 ± 0.04b	7.42 ± 0.03d	8.15 ± 0.04b	8.19 ± 0.02b	10.05 ± 0.11a
Total starch (g/100gDW)	58.24 ± 0.38e	62.19 ± 0.42d	65.4 ± 0.47c	67.12 ± 0.41b	67.64 ± 0.40b	68.88 ± 0.54a
Protein (%)	12.32 ± 0.10d	12.91 ± 0.15b	12.59 ± 0.05c	12.04 ± 0.03e	13.16 ± 0.07a	13.03 ± 0.03ab
Crude fat (g/100gDW)	1.18 ± 0.12a	1.16 ± 0.14a	1.17 ± 0.14a	1.26 ± 0.11a	1.27 ± 0.22a	1.24 ± 0.02a
Crude fibre (%)	—	—	—	—	—	0.30 ± 0.10
Ca (mg/L)	13.41 ± 0.15c	14.14 ± 0.09b	13.95 ± 0.24b	13.37 ± 0.07c	12.16 ± 0.07d	14.65 ± 0.08a
Fe (mg/L)	1.85 ± 0.03a	1.48 ± 0.02c	1.16 ± 0.01d	0.63 ± 0.00e	0.39 ± 0.01f	1.80 ± 0.03b
Mn (mg/L)	0.54 ± 0.01ab	0.47 ± 0.00c	0.52 ± 0.02b	0.56 ± 0.02a	0.54 ± 0.00ab	0.54 ± 0.01ab
Zn (mg/L)	1.21 ± 0.02a	1.18 ± 0.01b	1.16 ± 0.03b	1.15 ± 0.02b	0.99 ± 0.00c	1.24 ± 0.01a
Cu (mg/L)	0.20 ± 0.00a	0.20 ± 0.00a	0.19 ± 0.00b	0.20 ± 0.01a	0.19 ± 0.00b	0.20 ± 0.00a

^1^ data are expressed as the mean ± standard deviation. Values in the same row with different letters are significantly different (*p* < 0.05).

## Data Availability

Not applicable.
